# Dynamic regulation of hypoxia-associated regulatory CD39^+^CD73^+^ and IL35^+^ B cells in pancreatic cancer

**DOI:** 10.3389/fimmu.2026.1814123

**Published:** 2026-09-21

**Authors:** Hannah R. Hall, Jeffrey Song, Nancy Kren, Yuliya Pylayeva-Gupta

**Affiliations:** 1 Department of Pathology and Laboratory Medicine, School of Medicine, University of North Carolina at Chapel Hill, Chapel Hill, NC, United States; 2 Lineberger Comprehensive Cancer Center, School of Medicine, University of North Carolina at Chapel Hill, Chapel Hill, NC, United States; 3 Department of Genetics, School of Medicine, University of North Carolina at Chapel Hill, Chapel Hill, NC, United States

**Keywords:** adenosine, CD39, CD73, hypoxia, IL-35, pancreatic cancer, regulatory B cell

## Abstract

B cells can suppress anti-tumor T cell responses, representing a potential mechanism of resistance to immunotherapy. Multiple Breg subsets have been described in humans and mice. Bregs inhibit anti-tumor immunity through cytokine secretion, immune checkpoint expression, and potentially adenosine generation, which collectively suppress effector T cells, and strengthen immunosuppressive signaling. Hypoxia is a hallmark of pancreatic ductal adenocarcinoma (PDAC) and contributes to immune dysregulation within the tumor microenvironment (TME); however, the effects of hypoxia on B cell subsets remain poorly defined. In this study, we investigated how hypoxic stress shapes regulatory B cell phenotype using murine PDAC models. We show that nearly half of intratumoral B cells are hypoxic, which also increased across systemic B cell compartments with tumor burden. Hypoxic B cells were enriched for CD39 and CD73 ectonucleotidases and suppressed T cell activation via adenosine receptor–dependent mechanisms. Hypoxia preferentially induced CD39^+^CD73^+^ expression in peritoneal-derived B cells, indicating tissue-specific regulation, while splenic B cells showed limited responsiveness ex vivo. Conversely, hypoxia reduced IL-35 production by splenic Bregs through decreased Ebi3 expression and reduced activation marker levels. Within tumors, hypoxia correlated with CD11b^+^ and CD39^+^CD73^+^ B cells but not IL-35–producing subsets. Mechanistically, Ebi3 loss enhanced CD39 expression under hypoxia, whereas recombinant IL-35 suppressed CD39^+^CD73^+^ induction, suggesting reciprocal regulation between IL-35 and adenosine pathways. These findings reveal hypoxia-driven plasticity in immunosuppressive B cell programs in PDAC, characterized by suppression of IL-35–producing Bregs and promotion of adenosine-generating CD39^+^CD73^+^ B cells, highlighting potential therapeutic targets to modulate tumor-associated immunosuppression.

## Introduction

Pancreatic ductal adenocarcinoma (PDAC) is an aggressive malignancy that comprises 90% of pancreatic cancer cases and is typically diagnosed at late stages, resulting in a dismal 13% 5-year survival rate (American Cancer Society’s Cancer Facts & Figure 2023). In addition to therapeutic limitations associated with late detection, the PDAC tumor microenvironment (TME) is a treatment resistant landscape characterized by dense desmoplasia, poor vascularization, and heterogenous hypoxia—qualities/features that negate the efficacy of clinically approved therapies (1–4). Not surprisingly, the PDAC TME is considered immune “cold,” where exhausted T cells, regulatory B cells (Bregs), and suppressive myeloid populations work to suppress anti-tumor responses and bolster tumor viability (5–7).

Diverse Breg subsets have been described in both human and murine systems. Mechanistically, Bregs suppress anti-tumor immunity through multiple pathways, most prominently via secretion of immunoregulatory cytokines such as IL-10, IL-35, and TGF-β, which inhibit effector T cell activity, promote regulatory cell differentiation, and reshape inflammatory signaling; expression of immune checkpoint molecules including PD-1, PD-L1, and TIM1 that directly limit T cell function, with some reports of production of adenosine, which may suppress cytotoxic immune responses via adenosine receptors within hypoxic tumors (8, 9). Our group showed that IL35^+^ regulatory B cells directly promote the progression of PDAC in preclinical models, in part by suppressing effector CD8^+^ T cell activity (10, 11). This Breg subset accounts for only a fraction of TIL-B, showcasing how a minority of B cells can shape tumor fate and underscoring the need to define additional Breg phenotypes in PDAC. Understanding how the TME shapes Breg differentiation and function is critical for identifying prognostic biomarkers and for designing strategies that enhance beneficial B cell responses while mitigating Breg-mediated immunosuppression to improve cancer immunotherapy outcomes.

Though hypoxia has been described in PDAC for decades, hypoxia and its expansive web of associated stress response pathways have only recently emerged as potential therapeutic targets (2, 12–16). PDAC tumors with transcriptionally “hypoxia-high” signatures correlate with worse patient survival and reduced T cell, natural killer cell, and antigen-presenting dendritic cell cytotoxicity and abundance compared to “hypoxia-low” tumors (15). Strategically targeting PDAC therefore mandates a better understanding of the mechanisms behind hypoxia-driven immune dysfunction and suppression. While the impact of hypoxia and its primary transcription factor hypoxia inducible factor 1 alpha (HIF1α) on B cell homeostatic maturation has been well studied, the impact of hypoxia on mature B cells, especially in the context of cancer, remains understudied. Given that hypoxia and HIF1α are thought to upregulate CD39 and CD73, enzymes responsible for hydrolyzing ATP into adenosine, we hypothesized that some TIL-Bs contribute to the pool of hypoxia-associated adenosine in PDAC via hypoxia-mediated upregulation of CD39 and CD73 (17–20). This study investigates how hypoxia and B cell origin may impact the subsequent regulatory B cell fate in cancer.

## Materials and methods

### Mouse models

All mice were housed in a pathogen-free facility and used in compliance with protocols approved by the Institutional Animal Care and Use Committee of the University of North Carolina at Chapel Hill. Both male and female mice aged 8–12 weeks were used, and experiments were designed to include age-matched littermate controls. Wildtype *C57BL/6* mice were purchased from The Charles River Laboratories (strain #027). *Ebi3-/-* (#008691), CD45.1 C57BL/6 mice (strain #002014), and µMT (stock #002288) mouse strains were purchased from Jackson Laboratories and maintained at UNC. The *Kras^LSL-G12D/+^;Trp53^LSL-R172H/+^; PDX1^Cre/+^* (*KPC*) mice have been described previously (21). IL35 reporter mice were generated as described previously (22). Briefly, Ebi3^Tom/Tom^ mice gifted from D. A. Vignali (University of Pittsburgh, Pittsburgh, PA) were crossed with *IL12a^GFP/GFP^* mice generated at the University of North Carolina at Chapel Hill (Chapel Hill, NC, USA) to yield progeny homozygous for both reporter alleles. *CD19^Cre^;Ebi3^L/L^* mice were generated by crossing CD19^Cre^ mice to *Ebi3^Tom.L/L^* mice in our colony for two generations to obtain homozygosity at Ebi3 locus (23). Resulting mice lack expression of Ebi3 in B cells (*B^Ebi3-/-^*) (10).

### Cell lines and orthotopic tumor modeling

The murine PDAC cell line *KPC4662* was derived from primary pancreatic tumors of C57Bl/6J *KPC* mice by Dr. Vonderheide’s laboratory (24). Cells were maintained at 37 °C and 5% CO2 in complete DMEM and were confirmed to be Mycoplasma and endotoxin free. Cells were used at <16 passages. For intrapancreatic injection of cancer cells, mice were anesthetized using a ketamine (100 mg/kg) and xylazine (10 mg/kg; Med-Vet International) cocktail. 75,000 KPC cells in ice-cold PBS mixed at 1:1 dilution with Matrigel (Cat# 354234, Corning) were injected into the tail of the pancreas. To detect hypoxic cells/regions, we I.P. injected pimonidazole (60mg/kg) 45–90 minutes prior to euthanasia (mice were euthanized via CO_2_ at 2.8 L/min followed by cervical dislocation) of *KPC*, *KC* mice and/or orthotopically injected KPC mice (*oKPC)*. Of note, because these adducts are stable following reoxygenation, pimonidazole captures cells with a recent history of hypoxia, an important feature for applications such as flow cytometry that require prolonged sample processing. For adoptive transfer experiments, total splenic B cells were isolated from CD45.1 WT mice using negative magnetic selection (EasySep Mouse B cell Isolation Kit, #19854, StemCell Technologies). Two million live purified live B cells were intravenously injected into CD45.2+ mice via tail vein in 100µL sterile PBS one day prior to orthotopic tumor implantation. For all cell enumeration, Trypan Blue staining was used with the Countess Automated Cell Counter (Invitrogen AMQAX2000). Unless otherwise indicated, experiments were performed using 8-12week-old mice of indicated genotypes with at least 3–6 mice per group in triplicate.

### Histology and image analysis

Tissues collected for histology were fixed in 10% formalin for at least 48 hours at 4C^°^, then transferred to 70% EtOH. Paraffin embedding was performed by the Pathology Services Core at University of North Carolina at Chapel Hill. Five-micron FFPE sections were rehydrated via ethanol gradient and underwent sodium citrate antigen-retrieval before being incubated with rabbit anti-CD19 (1:200 Cat# 90176S, Cell Signaling Technologies), rat anti-pimonidazole (1:50 Cat# Rat-mAB, Hypoxyprobe) or rabbit anti-pimonidazole (1:200 Cat# R2710-B., Hypoxyprobe). Vectastain ABC Kit, Peroxidase (Standard) (Cat# PK-4000, Vector Laboratories), DAB (3,3’-diaminobenzidine) Substrate Kit (Cat# SK4100, Vector Laboratories), Vectastain ABC-AP Kit, Alkaline Phosphatase (Standard) (Cat # AK-5000, Vector Laboratories), and ImmPACT Vector Red Substrate Kit, Alkaline Phosphatase (Cat# SK-5105, Vector Laboratories) were used to detect antibodies per manufacturer protocols. Slides were counterstained with Harris hematoxylin (Sigma), dehydrated, and mounted with DPX mounting media (Cat# 06522, Sigma). Images were acquired using Nikon Eclipse Ni-U microscope with NIS-Elements software (Nikon) or VS200 Slide Scanner at the Hooker Imaging Core Facility (University of North Carolina at Chapel Hill). QuPath v0.6.0 (Github) was used to quantify the percent of tumor tissue that was hypoxic. On each measured tissue, a mask was applied to detect and exclude blank space from total tissue area. The threshold for detecting hypoxic tissue was established by verifying QuPath could accurately detect Hematoxylin vs IHC stain and then overlaying a detection threshold that matched the IHC signal. Masks and thresholds were standardized using one representative tumor section then applied to all images.

### Flow cytometry

To prepare single-cell suspensions for surface staining, plungers from 5mL syringes were used to smush spleens through a 70µM filter fitted atop 50mL conicals, washed through with ice cold PBS, pelleted at 300xg for 5min, resuspended in RBC lysis buffer for 5 mins, 20mL PBS added and then tubes spun again, resuspended in 1mL FACS buffer and filtered through 70µm filter once more. Tumor tissues were finely minced with razor blades before incubating on a 37°C shaker for 20 minutes in a solution of RPMI, Collagenase Type IV, DNase, and Soybean Trypsin Inhibitor, as described previously (25). The solution was poured through a 70µM filter, spun down and resuspended in 7mL PBS that was carefully layered atop 7mL of Ficoll in a 15mL tube and spun at 2,400rpm for 24min (ramp up and down both set to 0). The cloudy middle layer was collected, spun down, and washed once with PBS. Blood was collected in purple top tubes via post-mortem eye bleed. Blood was transferred to 15mL conicals, mixed with RBC lysis buffer, incubated for 5 minutes, PBS added, and tubes spun down at 300xg for 5 minutes. The resulting pellet was washed once in PBS. Single-cell suspensions of cells from each tissue were plated in round-bottom 96 well plates, washed once more with PBS, and stained with a viability dye for approximately 10 minutes at room temperature, protected from light. Homemade FACS buffer (500mL Gibco DPBS, 2mL 0.5M EDTA, 2.5g bovine serum albumin) was used for all washes prior to any fixation methods. Prior to surface staining, FACS-washed cells were resuspended with 10% TruStain FcX block (Cat# 156604, Biolegend) in FACS (50uL per well) for 5 minutes followed by 1:1 addition of Brilliant Staining Buffer (Cat# 566349, BD) containing pre-titrated fluorophore-conjugated antibodies (**Supplementary Table 1**) for 20 minutes at room temperature, protected from light. All flow cytometry samples are also stained with Live/Dead stain and only live cells are further analyzed. Importantly, all experiments including IL35 reporter cells remained unfixed to preserve endogenous GFP and TdTomato signals. For all other experiments, surface-stained cells were incubated with Fixation Buffer (Cat# 420801, BD) for 10 minutes at room temperature, protected from light. For experiments requiring cytoplasmic or nuclear detection of proteins, we proceeded with the FoxP3 Transcription Factor Staining Buffer Kit (00-5523-00, Invitrogen), following the manufacturer protocol. Cells were acquired on a Cytek Aurora 5L spectral flow cytometer with SpectroFlo software (Cytek). Cytek 5L Aurora daily QC was performed using SpectroFlo QC beads (Lot 2004) each day prior to use to ensure optimal laser performance. Unstained single cell suspensions for tumor and spleen were used to establish autofluorescence during unmixing in SpectroFlo. Both UltraComp eBeads plus (cat# 01-3333-42, Invitrogen) and tissue-derived single cell suspensions were used to generate single stain controls and were remade at minimum after every laser recalibration by a Cytek technician. For all *ex vivo* and *in vivo* experiments, data was analyzed using OMIQ (Dotmatics), where each sample was gated on forward vs side scatter, singlets, live cells, then CD45^+^ cells, followed by indicated stains.

### Isolation of leukocytes for *ex vivo* experimentation

For peritoneal B cells, 8mL ice cold FACS buffer I.P. was injected through a 27g x ½ needle (BD PrecisionGlide, Cat. 305109) and the inflated abdomen massaged for 1–2 minutes to dislodge cells. Most of the fluid was aspirated through a 25g x 1 needle (BD PrecisionGlide, Cat. 305125), then a small incision was made while holding the body wall up with forceps to aspirate remaining fluid by bulb transfer pipette. Peritoneal fluid was stored on ice, spun (1,500rpm,10 min), the supernatant decanted and pellet resuspended in 1mL FACS buffer. For splenic-derived B cells, the spleen was collected, stored on ice in RPMI media, and smushed through a 70uM mesh strainer with a sterile 10mL syringe plunger and washed with ice cold PBS. Cells were spun (300xg, 5 min) and resuspended in 5mL RBC lysis buffer for 5 min at RT, topped with 35mL PBS, spun again, and the resulting pellet resuspended in FACS and passed through another 70µM strainer into a 5mL round bottom collection tube. The manufacturer instructions were followed for negative selection of pan-B cells via EasySep magnetic bead isolation (Cat# 19844, StemCell) and viable cells quantified by Countess via Trypan Blue staining. B cells were seeded at 1e5 cells/well (96 well plate), 5e5 cells/well (48 well plate), or 2e6 cells/well (6 well plate) in complete RPMI + 1x 2-β-mercaptoethanol (Cat #21985023, Gibco) containing αIgM, µ chain (10 µg/mL, Cat# 115-006-075, Jackson Immunoresearch), purified αCD40 (1 µg/mL, clone HM40-3, Cat# 102902, Biolegend), and/or LPS (2 µg/mL, Cat# L4391-1MG, Sigma) and cultured in normoxia (5% CO_2_, 21% O_2_) or hypoxia (5% CO_2_, 1% O_2_; Galaxy 48 R CO_2_ Incubator, New Brunswick, Eppendorf) at 37 °C. In certain experiments, B cells were treated with sodium L-lactate (L7022, Millipore Sigma) or oxamate (HY-W013032A, MedChem Express) at doses indicated in corresponding Figures. B cells were also treated with 50ng/ml rIL-35, AB680 (HY-125286, MedChem Express) or 20 mmol/L STA-21 (145648, Abcam).

For T cell and B cell co**-**cultures, 5e4 peritoneal B cells were added 1:1 to splenic T cells seeded in 96 well plates. T cells were prepared from healthy murine spleens as described above and negatively selected using EasySep magnetic bead isolation kit (Cat# 19851, StemCell) following the manufacturer’s instructions. Prior to seeding, isolated T cells were incubated with Cell Trace Violet (Cat# C34557, Thermo) following the manufacturer’s instructions. T cells were stimulated with CD3/CD28 magnetic beads following the manufacturer’s instructions (Cat# 11456D, Thermo), recombinant mouse IL-2 (Cat# 575408, Biolegend), and/or adenosine receptor 2a (A2ar) inhibitor SCH 58261 (Cat# HY-19533, MedChem), and/or adenosine (Cat# A4036-5G, Sigma). To enable cytokine detection, we added a Cell Activation Cocktail containing Brefeldin-A, PMA, and ionomycin (Cat# 423303, BioLegend) for the last five hours of culture.

To detect extracellular adenosine produced by B cells, conditioned supernatant was aliquoted into a black walled, clear bottom 96 well plate (Cat# M33089, Thermo) following the manufacturer instructions for Adenosine Detection Kit (Cat# MET-5090, CellBioLabs) and scanned using Cytation5 Imaging Reader (BioTek).

### Analysis of publicly available datasets

The publicly available Loveless et al. dataset (26) was downloaded through Zenodo (https://zenodo.org/records/14199536) and further evaluated in R (4.4.0). Once the preprocessed data was downloaded from Zenodo, Seurat’s (v5.5.1) subset function was utilized to select for only cells in the B cell cluster. Seurat’s FeatureScatter was then used to display CD39 and CD73 on a scatter plot split by the disease state (adjacent normal, primary tumor or metastatic lesion) and grouped by sample. To evaluate the percentage of B cells that this population represents Seurat’s subset was used to select B cells with a CD39 expression greater than 1 and CD73 expression greater than 1. The resulting CD39^+^CD73^+^ B cells were then calculated as a percent of the total B cells for each sample. The Ma et al. dataset contains scRNAseq data from purified B cells (27). This pre-processed dataset was downloaded from Zenodo (https://zenodo.org/records/11468564). The data was then subsetted by using Seurat subset to select for primary tumors. Seurat’s FeatureScatter was used to display CD39 and CD73 on selected cells. To evaluate the percentage of B cells that are positive for both CD39 and CD73 subset was used to select for B cells with CD39 expression greater than 1 and CD73 expression greater than 1. The resulting CD39^+^CD73^+^ B cells were then calculated as a percent of the total B cells for each sample. The graphs was generated in Prism, and statistical analysis was done using Welch’s t-test (26).

### Statistics

All statistical analyses were conducted in Graphpad Prism version 10.6.0 for Windows 64-bit. One-way Anova was used when more than 2 variables were being compared at once, while paired or unpaired Student’s T test was used to compare two groups. The tests used in each Figure are stated in the Figure Legends. NS = not significant; *p-value<0.05, **p-value<0.01, ***p-value<0.001, ****p-value<0.0001.

## Results

### Intratumoral B cells colocalize with zones of hypoxia

To understand how B cells are impacted by hypoxia in PDAC, we used a syngeneic orthotopic injection model where murine pancreatic cancer cells featuring endogenous expression of oncogenic Kras and Tp53 (*LSL-Kras^G12D/+^;LSL-Trp53^R172H/+^;p48^Cre/+^*)(*KPC*) are injected into pancreata of C57Bl6J mice (orthotopic *KPC*, *oKPC*) (24). Alternatively, we used transgenic *KPC* mice (*LSL-Kras^G12D/+^;LSL-Trp53^R172H/+^;Pdx1^Cre/+^*), a genetically engineered model of spontaneous PDAC formation (21). To specifically detect hypoxic cells, we injected *oKPC* or *KPC* mice with pimonidazole (Pimo), a 2-nitromidazole compound that forms covalent adducts with thiols in proteins, peptides, and amino acids in hypoxic settings for detection by anti-pimonidazole antibodies (**Figure 1A**) (28–30). Of note, Pimo labels viable cells currently experiencing hypoxia as well as those with a recent history of hypoxic exposure. Using QuPath based quantification of Pimo-positive regions, we observed significant increase in total hypoxic tissue across both PDAC models analyzed, with *KPC* tumors showing the greatest percent positive area for pimonidazole compared to *oKPC* tumors, possibly because of the more desmoplastic nature of that model (**Supplementary Figures 1A–E**; **Figures 1B, C**). Visually, the pimonidazole staining patterns across all murine tumor models (and the absence of staining in normal-adjacent regions) resembled a previous report of pimonidazole staining in resected PDAC tumors and normal adjacent tissue (NAT) from 10 patients, suggesting that the hypoxic pattern in murine PDAC models mimic those found in patients (**Figure 1B**) (3). To detect hypoxic regions that overlap with infiltrating B cells, we stained sections by double IHC and were able to identify overlapping CD19^+^ B cells (brown DAB stain) and Pimo^+^ hypoxic (Chromagen Red stain) regions in both *oKPC* and spontaneous *KPC* models (**Figures 1D–F**).

**Figure 1:**
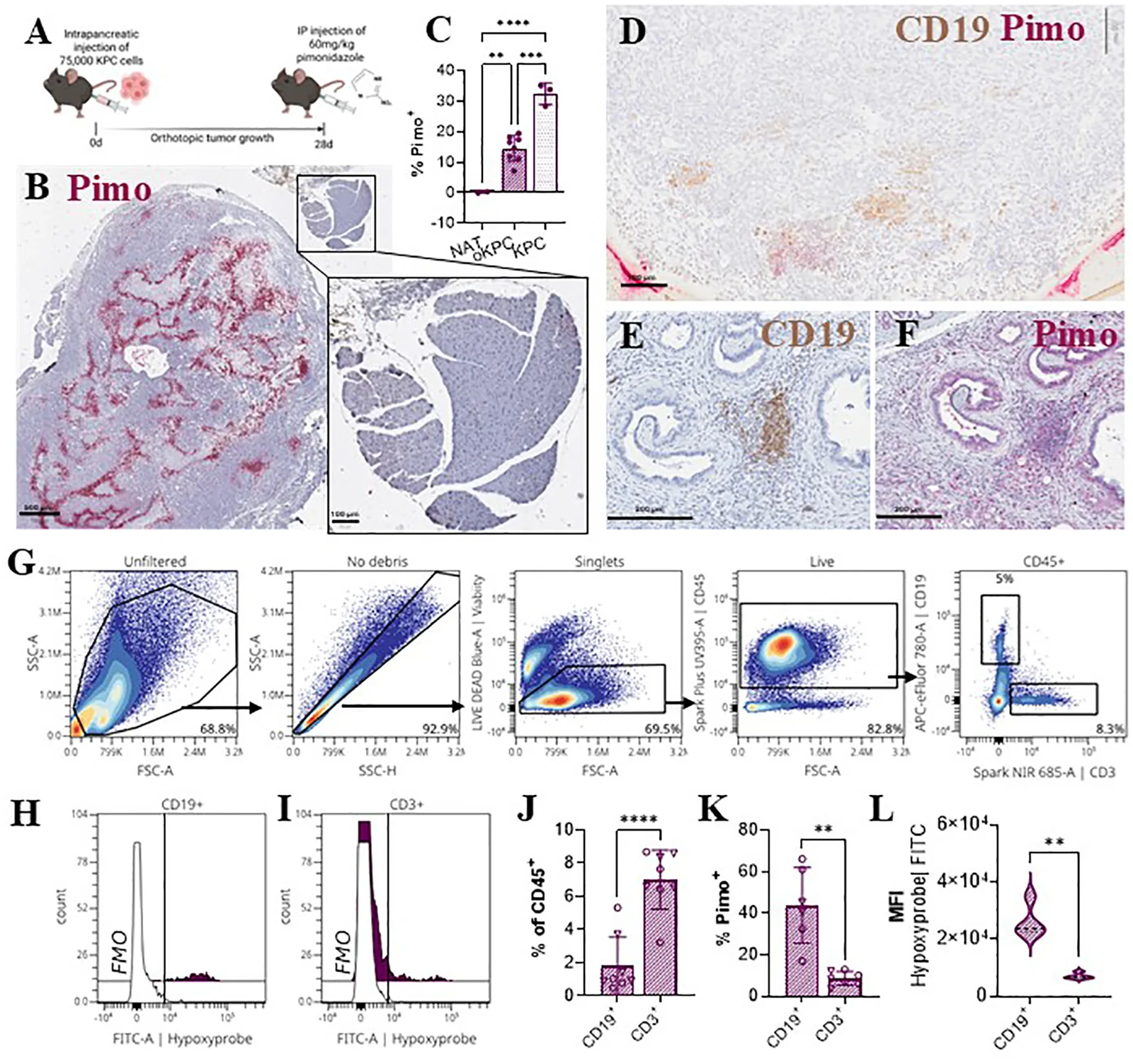
High proportion of tumor-infiltrating B cells are pimonidazole-positive. **(A)** Schematic overview of experimental procedure for the induction and collection of pimonidazole-labeled *oKPC* tissues. **(B)** Representative image of Pimo staining pattern in an *oKPC* tumor section (scale bar, 500 µm) with inset highlighting the absence of stain in normal adjacent pancreatic tissue (NAT) (scale bar, 100 µm). **(C)** Quantification of percent positive area for total Pimo staining NAT, *oKPC*, and spontaneous *KPC* tumor sections. Each point represents one tumor section. **(D)** Dual IHC on an *oKPC* tumor section stained for Pimo (red) and CD19 (brown; scale bar, 100 µm). **(E, F)** Single IHC for CD19 **(E)** or Pimo **(F)** on serial sections of a KPC tumor (scale bars, 200 µm). **(G)** Representative gating strategy used to identify live CD45^+^ B cells and T cells isolated from oKPC tumors. **(H, I)** Representative histograms of Pimo staining for total intratumoral CD19^+^ **(H)** or CD3^+^ **(I)** lymphocytes (purple) compared to tumor-specific, respective cell type FMOs (white). **(J)** Quantification of TIL-B vs TIL-T as a percent of CD45^+^ cells. **(K)** Quantification of Pimo^+^ T or B cells (two representative cohorts pooled, designated by open triangle or open circle). **(L)** The intensity of Pimo staining form **(K)** as determined by geometric mean fluorescence intensity (MFI). n=6-8. Error bars indicate SEM. P-values were calculated using a One-way Anova **(D, E)** or paired t-test **(J–L)**. ns, not significant; *p<0.05, **p<0.01, ***p<0.001, ****p<0.0001.

To better quantify hypoxic tumor-infiltrating lymphocytes (TILs), we used spectral flow cytometry to detect and quantify TIL-B cells (**Figure 1G**). The frequency of live Pimo^+^CD45^+^ cells compared to total CD45^+^ cells was determined by comparison to FMO samples (**Figures 1H, I**). Despite B cells only comprising ~5% of the TIL population, nearly half were hypoxic, whereas only ~9% of intratumoral T cells were hypoxic (**Figures 1J, K**). Notably, *all* hypoxic B cells had a brighter pimonidazole signal than hypoxic T cells (**Figure 1L**). These findings underscored our endeavor to further characterize B cell populations that are susceptible to hypoxia.

Accumulating evidence suggests that recirculation of immune cells from the tumor bed into surrounding vascular bed and tissues may The systemic impacts of tumor hypoxia are not well understood, with limited evidence linking local tumor hypoxia with anemia and bone marrow metastasis and implicating hypoxic, tumor-derived exosomes in facilitating the formation of pre-metastatic niches (31, 32). We therefore set out to establish whether we can detect changes in lymphocyte hypoxia levels across tissues of healthy versus tumor-bearing *oKPC* mice. We observed a significant increase in the frequency and overall intensity of Pimo MFI in total splenic hypoxic CD45^+^ leukocytes (**Supplementary Figures 2A, B**). The overall percentage of splenic B cells increased slightly, however, the frequency of splenic hypoxic B cells remained steady with significant increase of Pimo^+^ MFI (**Supplementary Figures 2C–E**). The same trends were true for total lymphocytes and B cells in the peripheral blood (**Supplementary Figures 2F–I**). Interestingly, draining lymph nodes did not exhibit any tumor-associated changes in lymphocyte or B cell hypoxia (**Supplementary Figures 2J–M**). This data suggests that tumor-associated increases in hypoxia may impact diverse systemic pools of B cells.

### Hypoxic B cells are preferentially enriched for dual positive CD39^+^CD73^+^

Hypoxia is thought to contribute to immunosuppressive properties of TME at least in part by driving the expression of ectonucleotidases CD39 and CD73, together hydrolyzing canonically inflammatory ATP into immunosuppressive adenosine (20). To understand if B cells that have been exposed to hypoxia upregulate ectonucleotidases, we profiled Pimo^+^ or Pimo^-^ B cells for expression of CD39 and/or CD73. Indeed, nearly 80% of Pimo^+^ TIL B cells (TIL-B) expressed CD39, with ~50% co-expressing CD73 and this expression pattern was significantly enriched in Pimo^+^ B cells (**Figures 2A-C, H**). Both splenic and circulating hypoxic B cells were also significantly enriched for CD39 expression, though the incidence of dual CD39 and CD73 expression among these cells did not exceed ~15% (**Figures 2C–E**). We further characterized hypoxic peritoneal B cells from *oKPC* mice and found a significant enrichment pattern of CD39^+^ only or dual CD39^+^CD73^+^ in hypoxic peritoneal B cells from tumor-bearing mice (**Figure 2F**). Across all tissues from tumor-bearing mice, there were no hypoxia-associated differences in the abundance of CD73^+^ only B cells (**Figures 2B–F**), indicating that hypoxia predominantly associates with CD39 expression in this population. Respective viability data for the above experiments is shown in **Figures 2H, I**. Overall, these data indicate that B cells experiencing hypoxia are significantly enriched for upregulation of CD39 and CD73 on B cells.

**Figure 2:**
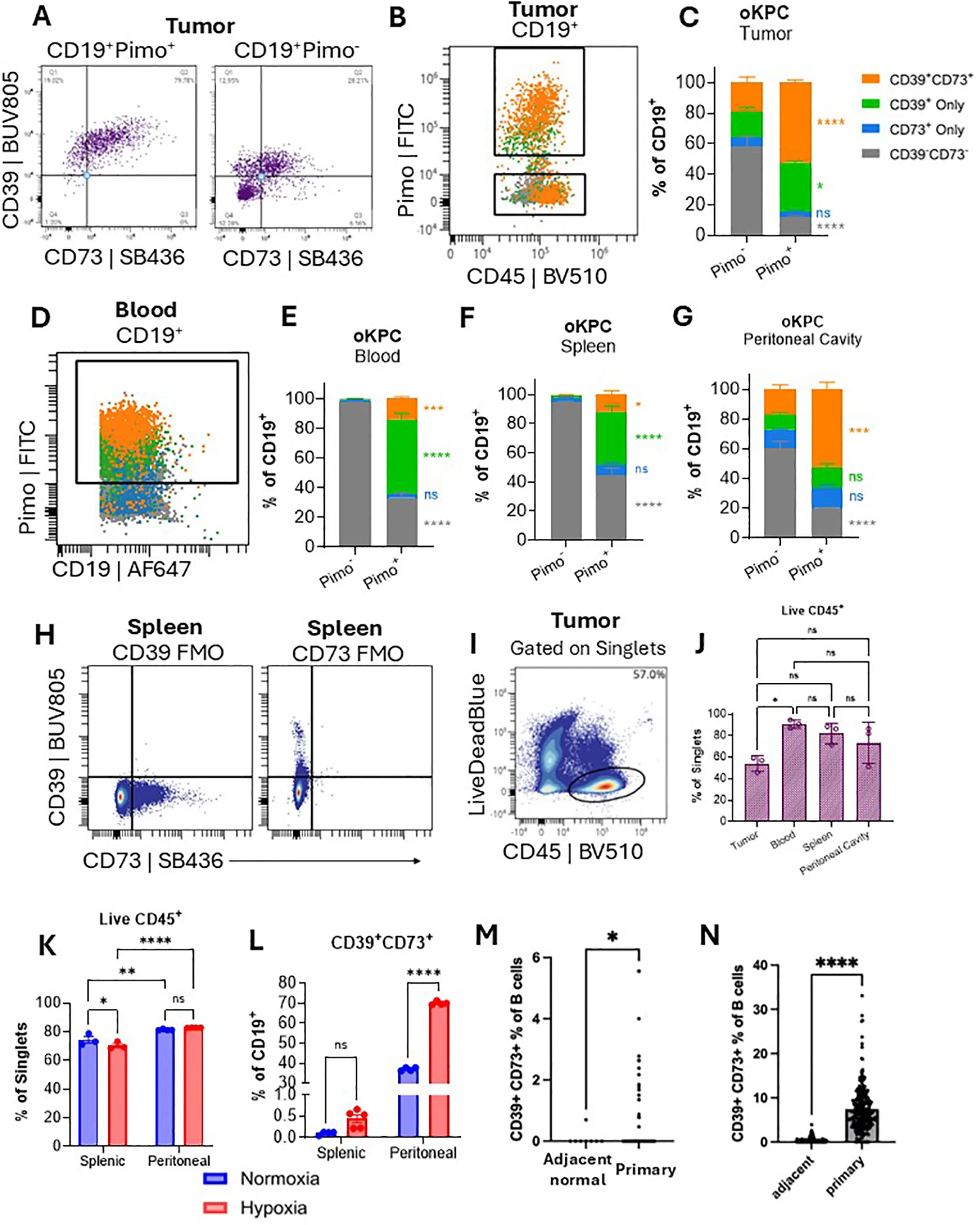
Pimo^+^ oKPC B cells are preferentially enriched for dual positive CD39^+^CD73^+^. **(A)** Representative flow plots depicting CD39^+^CD73^+^ staining gated from the Pimo^+^CD19^+^ B cells (left) and Pimo^-^CD19^+^ B cells (right). **(B)** An overlay of dual positive CD39^+^CD73^+^ (orange), CD39^+^CD73^-^ (green), CD39^-^CD73^+^ (blue), and CD39^-^CD73^-^ (gray) staining on the CD45^+^Pimo^+^CD19^+^ gate (right). **(C)** Quantification of the distribution of dual positive, single positive, and dual negative CD39 or CD73 on Pimo^+^ compared to Pimo^-^ intratumoral B cells. **(D)** Representative overlay flow plot depicting the distribution of dual positive, single positive, or dual negative CD39 and CD73 staining in the Pimo^+^ CD19^+^ B cell gate from an *oKPC* blood sample. **(E–G)** Quantification of the distribution of dual positive, single positive, and dual negative CD39 or CD73 on Pimo^+^ compared to Pimo^-^ B cells in the blood **(E)**, spleen **(F)**, and peritoneal cavity of *oKPC* mice **(G)**. **(H)** Representative flow plots depicting CD39 (left) and CD73 (right) FMOs used to set the gates for **(E)**. **(I)** Representative flow plot depicting gating strategy for live CD45^+^ cells from a tumor tissue. **(J)** Percent of viable CD45^+^ cells across tissues. N = 3. **(K)** Percent of live CD45^+^ cells and **(L)** dual positive CD39^+^CD73^+^ B cells isolated from the spleen or peritoneal cavity, stimulated with LPS + anti-IgM, and cultured for two days under normoxia (blue) or hypoxia (red). **(M)** Frequency of ENTPD1(CD39) and NT5E (CD73) double positive B cells of total B cells in adjacent normal (n=8) or primary tumor (n=108) from the publicly available Loveless et al. pancreatic cancer data set (26). Each dot represents a patient sample. Out of 108 primary tumor samples only 25 had greater than 100 B cells that were identified in sequencing runs. Of those 25, 13 samples contained B cells that were double-positive for CD39 and CD73. **(N)** The expression of ENTPD1(CD39) and NT5E (CD73) on B cells in adjacent normal, primary tumor or metastatic lesions from Loveless et al. pancreatic cancer data set, each dot represents a cell, each color represents a patient (26). Error bars indicate SEM. P-values were calculated using a paired t-test **(C, E–G, M)** or two-way Anova **(J–L)**. NS, not significant; *P<0.05, **P<0.01, ***P<0.001, ****P<0.0001.

To understand how hypoxia drives expression of CD39 and CD73 on murine B cells, we cultured splenic- or peritoneal-derived B cells under hypoxia (1% pO2) or normoxia (21% pO2). Almost no splenic B cells were CD39^+^CD73^+^ after 48 hours of culture and the expression of CD39^+^CD73^+^ did not significantly increase under hypoxia (**Figures 2J, K**). Peritoneally derived B cells, however, had a higher baseline expression of CD39 and CD73 and were more responsive to hypoxia, exhibiting a nearly 2-fold increase in dual CD39^+^CD73^+^ (**Figures 2J, K**). Of note, it is understood that B cells in germinal centers (GCs) reside in physiologically hypoxic niches, and that Pimo^+^ B cells in this activated B cell subset may reflect normal GC biology rather than pathological tumor hypoxia. However, recovery of canonically defined CD95^+^GL7^+^IgD^-^ GC B cells from orthotopic KPC tumors is consistently below 1% of total cells (not shown) that we can isolate and are a challenging population to characterize. Thus, the overlap between Pimo^+^ B cells and GC compartment does not account for the majority of Pimo^+^ B cells that we are able to recover from tumors (**Figure 2B**). Interestingly, we do see an increase in the percent of Pimo^+^ GCs in the spleens of tumor-bearing mice compared to healthy mice (**Supplementary Figures 3A–C**), potentially corroborating a known elevated hypoxic GC phenotype (33). However, the frequency of Pimo^+^CD39^+^CD73^+^ splenic B cells is still significantly lower than the frequency of total Pimo^+^ B cells derived from spleens of oKPC mice. Together, these findings indicate that while a small subset of Pimo^+^ B cells likely reflects physiologic hypoxic germinal center biology, the majority of tumor-associated Pimo^+^ B cells represent a distinct hypoxic CD39^+^CD73^+^ B-cell population.

Finally, to determine whether human tumor-infiltrating B cells express ectonucleotidase machinery, we analyzed publicly available human PDAC single-cell RNA sequencing data (26), subsetting on the B cell compartment across donor, adjacent normal and primary tumor samples. Notably, double-positive *ENTPD1/NT5E* B cells were most apparent within the primary tumor samples, consistent with a tumor-enriched population of ectonucleotidase-expressing B cells (**Figure 2M**). Patient-level quantification of CD39^+^CD73^+^ B cells in the Loveless et al. pancreatic cancer dataset, demonstrated that this population is present across multiple independent patient samples and is not driven by a single patient or a technical feature of the dataset (**Supplementary Figure 3D**). We further validated these findings in an independent, B cell-enriched pan-cancer dataset from Ma et al. (27) which demonstrates the presence of CD39^+^CD73^+^ B cells across multiple cancer types and their enrichment in primary tumors compared with adjacent normal tissues (**Figure 2N**; **Supplementary Figure 3E**).

Previous work has shown that anti-inflammatory adenosine production by CD73^+^ B cells helps mitigate the severity of murine experimental colitis, while human CD39^+^ B cells were able to suppress T cell proliferation and cytokine production *ex vivo*, presumably via 5’-AMP synthesis (34, 35). To ascertain the capacity of CD39^+^CD73^+^ B cells to inhibit T cells via adenosine-mediated suppression, we cocultured freshly isolated peritoneal B cells and splenic T cells for 3 days. To confirm that *ex vivo* cultured B cells were proficient in producing adenosine under hypoxia, and did not lose expression of ectonucleotidases, we observed that B cells increased adenosine production and maintained dual CD39^+^CD73^+^ positivity after 3 days of culture, highlighting continuous expression of immunosuppressive markers on peritoneal B cells even in the absence of B cell-specific stimulation (**Supplementary Figures 4A–D**). To test adenosine-mediated suppression, we added an adenosine receptor 2a (A2ar) inhibitor SCH58261 to a subset of wells (36). All wells received CD3/CD28 beads and IL-2 to promote T cell activation and proliferation, and co-cultures were seeded 1:1 B:T cell ratio. Intracellular cytokine staining following a five hour incubation with Brefeldin Cocktail showed a marked increase in T cell IFNγ and TNFα (primarily in CD8^+^ T cells) with A2ar inhibition compared to no treatment and adenosine treatment (**Supplementary Figures 4E, F**). To validate CD73 as an enzymatic source of B cell-derived adenosine, we treated co-cultures with the CD73 inhibitor AB680 and observed increased CD4^+^ T cell activation (CD44^+^CD25^+^) comparable to SCH58261-treated cells (**Supplementary Figure 4G**). AB680 did not confer the same protective effect on CD8^+^ T cell TNFα and IFNγ production observed with A2aR inhibition, potentially reflecting compensatory adenosine generation via alkaline phosphatase- or adenosine kinase-mediated pathways that circumvent CD73 blockade. We also tested the ability of extracellular Adenosine (eAdo) and/or B cell co-culture to suppress T cell proliferation. Addition of eAdo potently suppressed proliferating T cells, as did co-culture with B cells, except when A2ar inhibitor was added (**Supplementary Figures 4H, I**). Thus, peritoneal-derived B cells could suppress T cell activation in an A2ar-dependent manner.

### Compartment-specific integration of hypoxia and glycolytic cues shapes the CD39^+^CD73^+^ B cell phenotype

Because lactate accumulation is a hallmark byproduct of hypoxia-driven glycolytic metabolism in solid tumors and has been implicated in HIF1α-dependent and lactylation-mediated induction of CD39 and CD73 in other cell types, we next asked whether treatment with lactate was sufficient to mimic or augment this hypoxic phenotype in B cells (37–39). In normoxia, exogenous L-lactate (10–40 mM) did not significantly alter CD39 or CD73 expression on peritoneal B cells, despite a dose-dependent increase in their viability, and thus did not recapitulate the hypoxia-induced CD39^+^CD73^+^ phenotype (**Supplementary Figures 5A–D**). By contrast, addition of L-lactate to hypoxia-cultured peritoneal B cells selectively increased total CD73 and dual CD39^+^CD73^+^ frequencies while modestly reducing total CD39, indicating that lactate can fine-tune, but not substitute for, hypoxia-specific signaling in establishing this adenosinergic phenotype (**Supplementary Figures 5A–D**). Splenic B cells treated with lactate exhibited a dose-dependent decrease in total CD39, total CD73, and dual CD39^+^CD73^+^ cells in both normoxic and hypoxic conditions, with minimal increases in viability, highlighting a distinct metabolic and regulatory program compared to peritoneal B cells (**Supplementary Figures 5E–H**).

To determine whether loss of lactate under hypoxic conditions could recapitulate the normoxic phenotype, we treated splenic and peritoneal B cells with a competitive inhibitor (oxamate) of lactate dehydrogenase (LDH), the enzyme responsible for catalyzing the conversion of pyruvate to lactate. At lower oxamate doses (5mM and 10mM), partial blockade of LDH leads to increased B cell viability of hypoxic and normoxic peritoneal B cells, as well as hypoxic splenic B cells, whereas the highest dose (30mM) is associated with decreased viability (**Supplementary Figures 5I, M**). Surprisingly, oxamate increased CD73 expression on hypoxic peritoneal and splenic B cells, as well as dual CD39^+^CD73^+^ expression on hypoxic peritoneal B cells, in a dose-dependent manner (**Supplementary Figures 5J, L, P**). CD39 expression decreased in response to LDH blockade, with hypoxic peritoneal B cells returning to normoxic CD39 levels and hypoxic splenic B cells dropping far below that of normoxic splenic B cell CD39 (**Supplementary Figures 5K,O**). Dual expression of CD39^+^CD73^+^ on hypoxic splenic B cells was not altered by oxamate, while normoxic splenic B cells exhibit a decrease to that of the hypoxic group (**Supplementary Figure 5N**).

Taken together, these findings demonstrate that hypoxia may be the primary driver of CD39^+^CD73^+^ induction in peritoneal B cells, with lactate and LDH activity acting as dose- and lineage-dependent modulators of ectonucleotidase balance and survival and further underscore a compartment-specific divergence in how splenic and peritoneal B cells integrate hypoxic and metabolic cues.

### Hypoxia decreases production of IL35 by B cells *ex vivo*

Our group has previously reported on a population of splenic-derived CD1d^hi^CD21^+^IL35^+^ regulatory B cells (Bregs) that directly promote tumor progression by, in part, suppressing CD8^+^ T cells (40, 41). Additional reports suggested that cytokine producing Bregs, such as IL10^+^Breg may expand in response to hypoxia (42). To understand how hypoxia impacts IL35-proficient B cells, we used an IL35 dual-reporter mouse line developed in our lab - a model designed to enhance detection of IL35’s two subunits by encoding TdTomato and GFP to detect Ebi3 and p35, respectively, such that double positive GFP^+^TdTom^+^ serve as a readout for IL35 expression (25). Isolated splenic B cells were then cultured under hypoxia or normoxia for 48 hours (**Figure 3A**). In line with previously published *ex vivo* IL35-reporter expression, LPS+αCD40 stimulation yielded the greatest population of IL35^+^ B cells (25). Strikingly, IL35 expression decreased under hypoxia compared to standard cell culture incubator normoxia across all stimulatory conditions (**Figures 3A, B**). Looking at the IL35 subunits individually, we observed approximately 50% decrease in TdTomato signal (Ebi3) in the αCD40+αIgM stimulation group cultured under hypoxia (**Figure 3C**), whereas GFP signal (p35), dropped by a modest, but significant, ~5% across all three stimulation groups (**Figure 3D**). Therefore, the hypoxia-driven decrease in reporter-measured IL35 production by splenic B cells might be attributed to a preferential decrease in the Ebi3 subunit. Interestingly, we observed similar trends towards decrease as opposed to increase of IL35 protein subunits, upon direct intracellular cytokine detection of p35 and Ebi3 in wildtype splenic B cells cultured with αCD40+αIgM for 4 days (**Supplementary Figures 6A–E**). In the αCD40-only stimulation group, hypoxia also decreased the frequency of B cells expressing activation marker CD86 (**Figure 3E**), while the MFI of CD86^+^ B cells decreased under hypoxia in the αCD40+αIgM or LPS groups (**Figures 3F, G**), suggesting that hypoxia impedes total splenic B cell activation. None of these cells were dual positive for CD39^+^CD73^+^ and had only miniscule positivity for either enzyme alone (**Figures 3H, I**).

**Figure 3:**
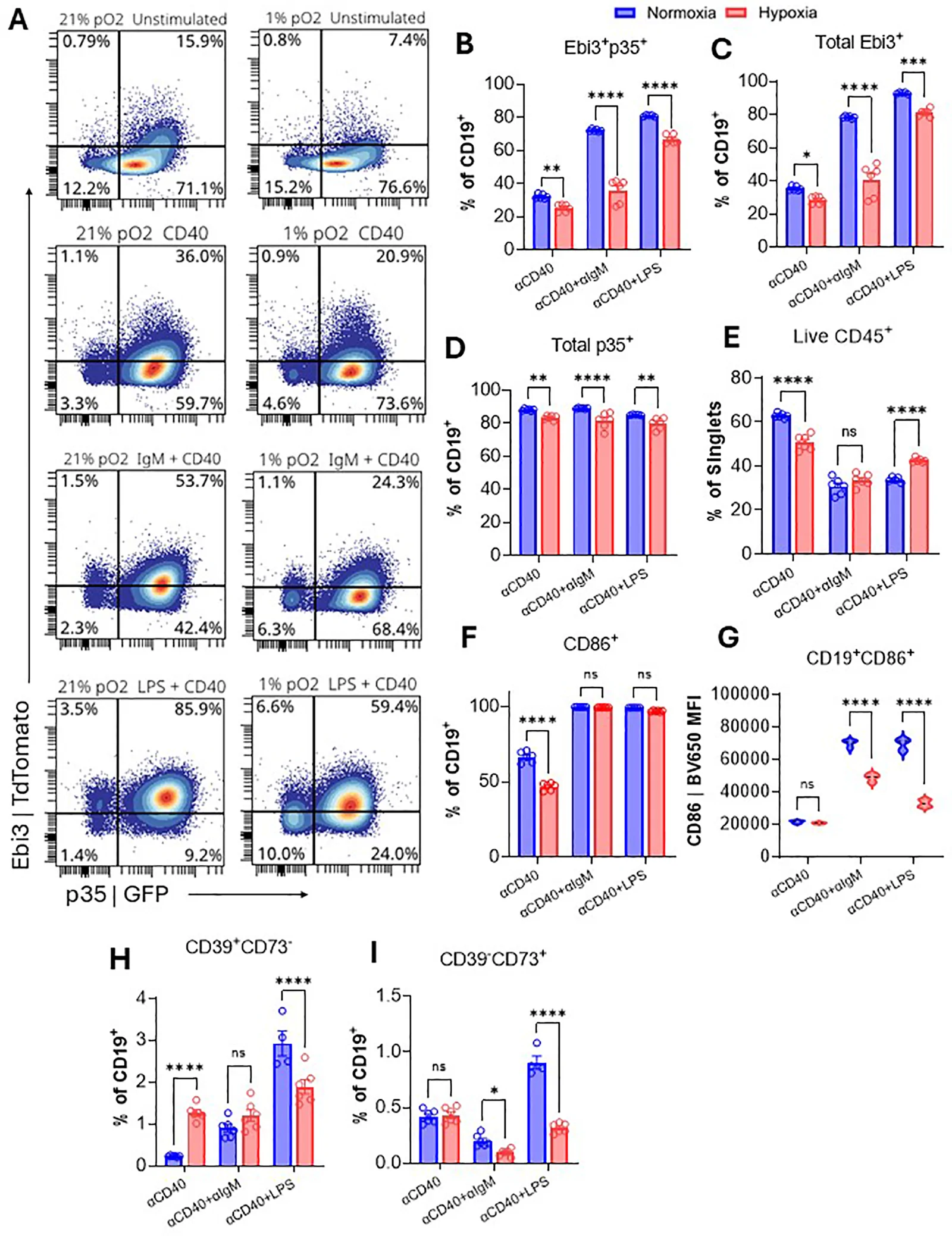
Hypoxia decreases production of IL35 by B cells *ex vivo.***(A)** Representative flow plots of IL35-reporter splenic B cells cultured for 2 days under normoxia (left column) or hypoxia (right column) in the presence of indicated stimuli. **(B)** Quantification of IL35^+^ (dual positive for GFP and TdTomato), **(C)** total Ebi3 (total TdTomato signal), or **(D)** total p35 (total GFP signal) on splenic B cells cultured in normoxia (blue bar) or hypoxia (red bar) for 4 days. **(E)** Quantification of Live CD45^+^ cells as a percent of singlets and **(F)** CD86^+^ as a percent of CD19^+^ B cells. **(G)** gMFI of CD86 stain gated on CD86^+^ B cells. **(H)** Quantification of CD39 and **(I)** CD73 expression as a percent of total CD19^+^ B cells. Error bars indicate SEM. P-values were calculated using a two-way Anova; NS, not significant; *P<0.05, **P<0.01, ***P<0.001, ****P<0.0001.

### Differential regulation of immunosuppressive B cells by hypoxia

Previously, our group used a tracing strategy to show that splenic B cells (potentially B-2 lineage), rather than peritoneal B cells (potentially B-1 lineage) preferentially expands into IL35^+^ Bregs (41). To understand where hypoxic CD39^+^CD73^+^ TIL-B may have originated from, we used published gating strategies to phenotypically stratify splenic-derived Bregs (CD19^+^CD21^+^CD1d^hi^) and peritoneally-derived B cells (IgD^-^CD21^-^CD11b^+^) from mice bearing orthotopic *KPC* tumors (43–46). As expected, ~10% of splenic B cells were positive for CD21^+^CD1d^hi^ markers compared to only 0.6% of peritoneal B cells (**Figures 4A, B**). Analysis of IL35 reporter signal and/or CD39, CD73 staining in splenic Bregs showed that these cells were enriched for IL35^+^ (50-60%), but not dual CD39^+^CD73^+^ expression (~12%; **Figures 4C, D**; **Supplementary Figure 7A**). We next confirmed that peritoneal-like CD11b^+^ B cells were largely absent from the splenic B cell pool (~.5%, percentage of grandparent CD19^+^ population) and enriched in the peritoneal pool (~30%; **Figures 4E, F**). Within this population, peritoneal B cells were enriched for dual CD39^+^CD73^+^ (~50%), but not for IL35 (~5%, **Figures 4G, H**; **Supplementary Figure 7B**). Peritoneal IgD^-^CD21^-^ lacking CD11b were not enriched for dual CD39^+^CD73^+^ or IL35^+^ (**Figures 4I–K**), highlighting the relevance of CD11b as a marker for dual positive CD39^+^CD73^+^ peritoneal B cells. Lastly, we report that although only <1% of peritoneal B cells were CD1d^hi^CD21^+^ (**Figures 4A, B**), that population contributes to both the IL35 and CD39^+^CD73^+^ pools (**Supplementary Figures 7C, D**). Similarly, <0.005% of splenic B cells were IgD^-^CD21^-^CD11b^+^ (**Figures 4E, F**), with about a fifth expressing both IL35 and CD39^+^CD73^+^ (**Supplementary Figures 7E, F**). Thus, there is a small overlap between these markers, which could potentially also indicate a degree of plasticity.

**Figure 4:**
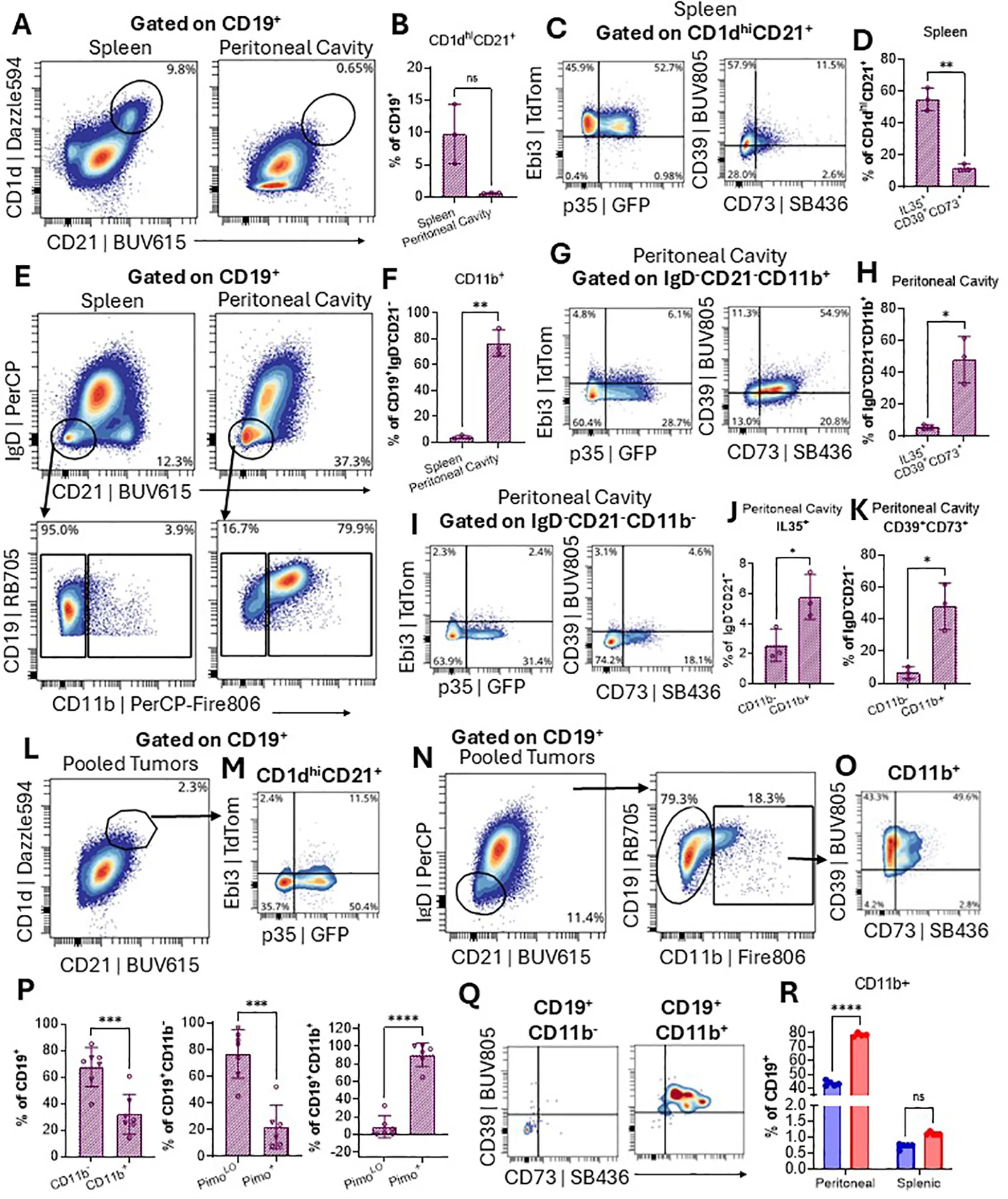
B cell responses to tumor-associated hypoxia are enriched in tissue specific manner. **(A)** Representative flow plots of CD1d^hi^CD21^+^ B cells from spleen (left) or peritoneal cavity (right) of an oKPC mouse. **(B)** Quantification of CD1d^hi^CD21^+^ B cells as a percent of total CD19^+^ B cells. N = 3. **(C)** Representative flow plots of Ebi3 (TdTomato) and p35 (GFP; left) and CD39 and CD73 (right) on splenic CD1d^hi^CD21^+^ B cells. **(D)** Quantification of IL35 and dual positive CD39^+^CD73^+^ as a percent of CD1d^hi^CD21^+^ B cells. N = 3. **(E)** Representative flow plots of IgD^-^CD21^-^CD11b^-^ or CD11b^+^ splenic B cells (left column) and peritoneal B cells (right column). **(F)** Quantification of IgD^-^CD21^-^CD11b^+^ B cells as a percent of total CD19^+^ B cells from **(E)**. **(G)** Representative flow plots of Ebi3 (TdTomato) and p35 (GFP; left) and CD39 and CD73 (right) on peritoneal IgD^-^CD21^-^CD11b^+^ B cells. **(H)** Quantification of IL35 and dual positive CD39^+^CD73^+^ as a percent of IgD^-^CD21^-^CD11b^+^ B cells. N = 3. **(I)** Representative flow plots of Ebi3 (TdTomato) and p35 (GFP; left) and CD39 and CD73 (right) on peritoneal IgD^-^CD21^-^CD11b^+^ B cells IgD^-^CD21^-^CD11b^-^ B cells. **(J)** Quantification of IL35 and **(K)** dual positive CD39^+^CD73^+^ on peritoneal IgD^-^CD21^-^ B cells based on CD11b status. N = 3. **(L–O)** Representative flow plots of TIL-B from pooled tumors. N = 5. **(L)** Gate for CD1d^hi^CD21^+^ TIL-B. **(M)** Ebi3 and p35 gated on CD1d^hi^CD21^+^ TIL-B. **(N)** IgD^-^CD21^-^CD11b^-^ or CD11b^+^ TIL-B. **(O)** CD39 and CD73 gated on IgD^-^CD21^-^CD11b^+^ TIL-B. **(P)** Quantification of CD11b positivity as a percent of total TIL-B (left) and quantification of Pimo^Lo^ vs Pimo^+^ on CD11b^-^ TIL-B (middle) and CD11b^+^ TIL-B (right). N = 7. **(Q)** Representative flow plots depicting CD11b^-^ (left) or CD11b^+^ (right) TIL-Bs on CD39 and CD73 gates. **(R)** Percent of CD11b^+^ B cells isolated from the spleens or peritoneal cavity, stimulated with LPS + anti-IgM, and cultured for two days under normoxia (blue) or hypoxia (red). Error bars indicate SEM. P-values were calculated using an unpaired t-test. ns, not significant; *P<0.05, **P<0.01, ***P<0.001, ****P<0.0001.

Next, we pooled five tumors from *oKPC* mice and applied CD39^+^CD73^+^ peritoneal Breg gating strategies (**Figures 4L–N**). Nearly 50% of IgD^-^CD21^-^CD11b^+^ Bregs were dual CD39^+^CD73^+^ (**Figures 4M, N**), consistent with the phenotype of peritoneal IgD^-^CD21^-^CD11b^+^ Bregs (**Figures 4H, K**). We then wanted to understand if TIL-B hypoxia correlated with CD11b^+^ B cell population. Across 7 tumors, an average 25% of TIL-B were CD11b^+^ and levels of hypoxia directly corresponded to CD11b status, such that ~80% of CD11b^+^ TIL-B were Pimo^+^, compared to only ~20% of CD11b^-^ TIL-B (**Figure 4O**). Indeed, hypoxic CD11b^+^ TIL-B expressed the greatest percentage (and MFI) of dual CD39^+^CD73^+^ (**Figures 4P–R**). Taken together, we determined that splenic CD21^+^CD1d^hi^ encompassed the majority of IL35^+^ Bregs, while peritoneal IgD^-^CD21^-^CD11b^+^ B cells were the primary source of dual CD39^+^CD73^+^ expression, drawing the correlation to this population as a Breg subset.

Finally, we adoptively transferred either CD45.2 peritoneal B cells into CD45.1 mice bearing 3.5 week orthotopic KPC tumors. At four weeks, we collected the tumors, spleen, tumor-draining lymph nodes, and peritoneal wash to understand where peritoneally-injected B cells trafficked (**Supplementary Figures 7G–S**). The transferred CD45.2 B cells accounted for 4.4% of B cells recovered from the peritoneal cavity, and 26% were IgD^-^CD21^-^CD11b^+^, while 11% of native CD45.1 B cells were IgD^-^CD21^-^CD11b^+^ (**Supplementary Figures 7G–K, S**). In the tumor, 2.7% of TIL-B were CD45.2^+^, demonstrating that peritoneal compartment B cells migrate to the pancreas in the presence of cancer (**Supplementary Figure 7L**). Notably, 11.5% of these cells exhibited the peritoneal-like Breg phenotype, compared to 3.8% of the native TIL-B population, while <0.5% of either CD45.1 or CD45.2 TIL-B were CD1d^hi^CD21^+^ (**Supplementary Figures 7L–S**). Thus, adoptively transferred peritoneal B cells trafficked to pancreatic tumors, where they were enriched for the peritoneal Breg phenotype compared with native tumor-infiltrating B cells, indicating that peritoneal B cells can migrate to the tumor microenvironment during pancreatic cancer.

To better understand if inhibition of CD73 impacts tumor growth in B cell dependent manner, we treated wildtype IL35 reporter and B cell-deficient (µMT) oKPC mice with AB680, a small molecule inhibitor of CD73 (**Supplementary Figure 8A**). Here we report AB680 modestly reduced tumor volume in IL35 reporter mice and nearly doubled the percentage of viable TILs compared to vehicle-treated wildtype mice and both µMT treatment groups (**Supplementary Figures 8B,C**). Notably, AB680 treatment lead to an overall increase in µMT tumor volume and also introduced a striking range of tumor sizes (**Supplementary Figure 8B**). AB680 treatment also significantly decreased the percentage of dual CD39^+^CD73^+^ TILs in wildtype mice compared to the vehicle group, whereas µMT TILs were less CD39^+^CD73^+^ at baseline and did not further reduce expression in response to AB680 (**Supplementary Figure 8D**). Collectively, these data are consistent with a model in which B cells contribute to the therapeutic effects of CD73 inhibition. The differential response to AB680 in wildtype and B cell-deficient mice suggests that B cell-associated CD73 supports an immunosuppressive tumor microenvironment and may contribute to PDAC progression.

### Loss of *Ebi3* enhances hypoxia-mediated CD39 expression ex vivo and rIL35 treatment rescues phenotype

Based on the observation that splenic IL35^+^ Bregs have negligible expression of either CD39 or CD73 *in vitro* and only minimally express dual CD39^+^CD73^+^ *in vivo* (**Figures 4C, D**), we wanted to understand whether IL35 may regulate a compensatory immunosuppressive mechanism via CD39^+^CD73^+^. To do this, we cultured WT or *Ebi3-/-* splenic B cells under normoxia or hypoxia with BCR and TLR ligands for five days and quantified the percent of dual CD39^+^CD73^+^ as well as the percent and MFI of CD39 only and CD73 only (**Figures 5A–E**). Ebi3-deficient B cells upregulated both CD39 and CD73, regardless of oxygen status, though hypoxia further increased CD39 and dual CD39+CD73+ expression (**Figures 5A–E**). Next, we wanted to determine if treatment with recombinant IL35 (rIL35) could rescue the elevated *Ebi3-/-* CD39^+^CD73^+^ phenotype. We also asked whether STAT3 activity is involved in mediating these phenotypes, since it is directly involved in IL35 receptor signaling (40). Under hypoxia, recombinant IL35 (rIL35) significantly reduced elevated CD39^+^CD73^+^ in *Ebi3^−^/^−^* B cells toward WT levels, driven primarily by a reduction in total CD73^+^, as total CD39^+^ was unaffected by rIL-35 and was instead reduced only by the STAT3 inhibitor STA21 (**Figures 5F-H**). In the combination treatment group, STA21 abrogated the suppressive effect of rIL35 on CD39^+^CD73^+^ and CD73^+^ under hypoxia, such that these populations remained elevated and comparable to untreated hypoxic *Ebi3^−^/^−^* B cells, while rIL-35 completely cancelled the STA-21-mediated reduction in CD39^+^ (**Figures 5F–H**). Interestingly, under normoxia, rIL-35 paradoxically elevated CD39^+^CD73^+^ on *Ebi3^−^/^−^* B cells, driven by increases in both total CD39^+^ and total CD73^+^, suggesting that IL-35 receptor signaling can promote ectonucleotidase expression when hypoxic cues are absent. Further, STA-21 was sufficient to block the rIL-35-mediated increase in CD39^+^CD73^+^ and total CD39^+^ under normoxia, and modestly decreased CD73^+^ expression (**Figures 5I–K**). Notably, STA-21 significantly reduced viability of both WT and *Ebi3^−^/^−^* B cells under hypoxia but not normoxia (**Supplementary Figures 9A,B**), suggesting that some of the observed reductions in CD39^+^ and CD73^+^ in STA-21-treated hypoxic cultures may partially reflect reduced cell survival in addition to signaling-mediated suppression of ectonucleotidase expression; rIL-35 alone did not significantly affect viability under either oxygen condition. Taken together, these data indicate that the oxygen environment determines the direction of IL-35 signaling on ectonucleotidase expression in Ebi3-deficient B cells, with hypoxia-specific suppression of CD73 driving the overall reduction in CD39^+^CD73^+^ in a STAT3-dependent manner, while normoxic IL-35 signaling instead promotes expression of both CD39 and CD73.

**Figure 5:**
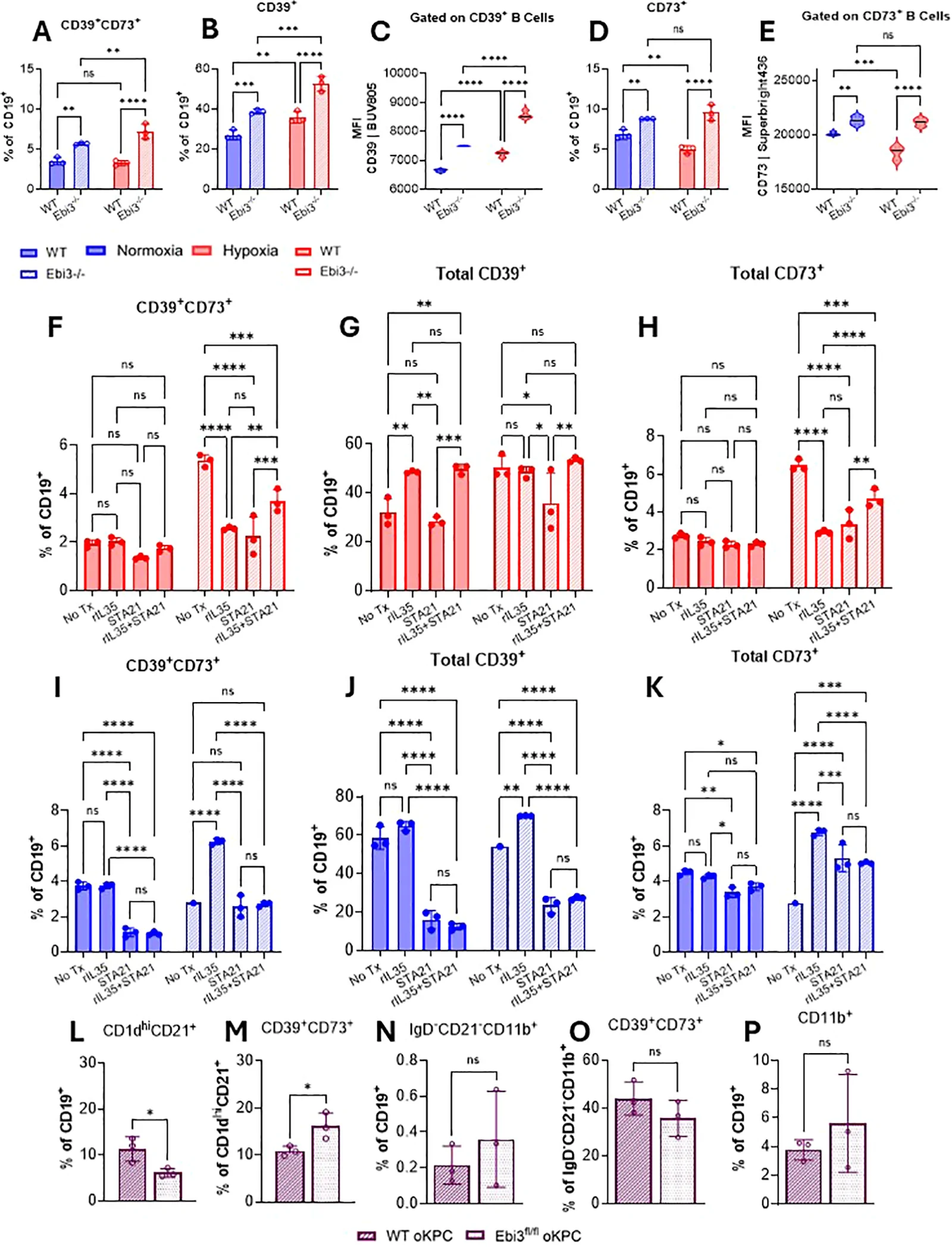
Loss of Ebi3 enhances hypoxia-mediated CD39 expression *ex vivo* and rIL35 treatment rescues this phenotype. **(A)** Quantification of dual positive CD39^+^CD73^+^, **(B)** percent CD39^+^, **(C)** CD39 gMFI, **(D)** percent CD73^+^, and **(E)** CD73 gMFI gated on splenic live B cells cultured with anti-IgM and LPS under normoxia or hypoxia for 5 days. **(F)** Quantification of dual positive CD39^+^CD73^+^, **(G)** CD39^+^, and **(H)** CD73^+^ on splenic live WT or *Ebi3-/-* B cells, cultured under hypoxia with the addition of recombinant IL35 (rIL35) and/or STAT3 inhibitor (STA-21). **(I)** Quantification of dual positive CD39^+^CD73^+^, **(J)** CD39^+^, and **(K)** CD73^+^ on splenic live WT or *Ebi3-/-* B cells, cultured under normoxia with the addition of recombinant IL35 (rIL35) and/or STAT3 inhibitor (STA-21). **(L–P)** Quantification of splenic B cells from WT healthy, WT oKPC, or *CD19-Cre;Ebi3^fl/fl^* oKPC mice. **(L)** Quantification of CD1d^hi^CD5^+^CD21^+^ B cells as a percent of total CD19^+^B220^+^ B cells. **(M)** Quantification of dual CD39^+^CD73^+^ on splenic CD1d^hi^CD5^+^CD21^+^ Bregs. **(N)** Quantification of IgD^-^CD21^-^CD11b^+^ B cells as a percent of total splenic CD19^+^B220^+^ B cells. **(O)** Quantification of dual CD39^+^CD73^+^ on splenic IgD^-^CD21^-^CD11b^+^ B cells. **(P)** Quantification of total CD11b^+^ splenic B cells. Error bars indicate SEM. P-values were calculated using a two-way Anova **(A–F)** or an unpaired t-test **(L–P)**. ns, not significant; *P<0.05, **P<0.01, ***P<0.001, ****P<0.0001.

Finally, to determine the relevance of this compensatory immunosuppressive phenotype *in vivo*, we profiled splenic B cells from WT healthy, WT *oKPC*, or *CD19^Cre^;Ebi3^fl/fl^ oKPC* mice (**Figures 5G, H**). We observed a significant 2-fold reduction in the frequency of splenic-like Bregs (CD1d^hi^CD21^+^), and a 2-fold increase in dual CD39^+^CD73^+^ B cells (**Figures 5L, M**). Importantly, loss of Ebi3 had no impact on the total percentage of peritoneal-like B cells in the spleen, nor the percent of dual CD39^+^CD73^+^ peritoneal-like cells (**Figures 5N–P**). These findings suggest that IL35 and CD39/CD73 may represent partially mutually exclusive regulatory programs, with a potential for reciprocal compensatory activation of either IL35 and/or adenosine-driven suppression programs in B cells.

## Discussion

Tumor-infiltrating B cells (TIL-Bs) can either support or undermine anti-tumor immune responses and correlate with positive or negative prognoses across solid cancers (5, 40). In the former role, effector B cells can secrete tumor-specific antibodies, present tumor-antigens to T cells, and release pro-inflammatory cytokines to facilitate sustained immune responses. In the latter role, regulatory B cells (Bregs) play a complex role in cancer immunity by contributing to the immunosuppressive tumor microenvironment (TME) that facilitates tumor progression despite ongoing immune surveillance. Early murine studies suggested a pro-tumorigenic role for B cells through suppression of CD8^+^ T cell responses, although subsequent findings indicate functional heterogeneity and context-dependent effects (40).

Hypoxia is a defining feature of pancreatic ductal adenocarcinoma (PDAC) and contributes to immune evasion through metabolic and transcriptional reprogramming of tumor-infiltrating cells. While hypoxia-driven immunosuppression has been extensively characterized in myeloid cells and T cells, comparatively little is known about how hypoxic stress shapes B cell function within tumors (47–50). Here, we demonstrate that intratumoral B cells can localize to hypoxic Pimo-positive niches and undergo subset-specific reprogramming that may drive distinct immunosuppressive mechanisms. Our findings reveal that hypoxia does not uniformly enhance all regulatory B cell (Breg) pathways but instead selectively promotes adenosine-producing CD39^+^CD73^+^ B cells in lieu of IL35-producing Bregs, highlighting previously unrecognized functional plasticity between splenic and peritoneal derived regulatory programs. An important limitation of this study is that pimonidazole reports the presence of hypoxic cells but does not distinguish between moderate and severe hypoxia. Consequently, we cannot determine whether the observed B-cell phenotypes are preferentially associated with adaptive metabolic rewiring characteristic of moderate hypoxia or with the stress responses elicited by more profound oxygen deprivation. Because our ex vivo studies were performed at 1% O2, the findings most closely model moderate hypoxia. Future studies using complementary approaches capable of resolving hypoxic gradients will be valuable for defining how oxygen availability differentially regulates regulatory B-cell function.

Developing B cells encounter a spectrum of oxygen gradients and hypoxic niches as they egress from the bone marrow (~2.5% pO2), enter circulation (~13.2% pO2), deposit in the spleen (1-5% pO2) or lymph node (~2% pO2), and encounter hypoxic light zones in germinal centers (GCs, <1% pO2) during antibody refinement (51). Studies in the contexts of normal physiology and autoimmune disease have leveraged B cell-specific deletion of HIF1α to interrogate the role of hypoxia in early B cell development and signaling. Loss of HIF1α stunted early-pre-B to pre-B cell differentiation in the bone marrow (but not pre-B to pre-pro-B and onwards), induced dysfunctional B1 peritoneal B cells, and led to increased levels of circulating autoantibodies (52, 53). In later stages of maturation, B cells oscillate in hypoxic light zones of GCs to proliferate, differentiate, and generate high-affinity, class-switched antibodies. However, it is unclear how aberrations in hypoxia or HIF1α might alter these mechanisms, with one study indicating constitutive activation of HIF1α in GCs leads to impaired maturation and class switching and another indicating that elevated hypoxia *improves* class switching, highlighting the complexity of this signaling matrix in later stages of B cell development (54–56). Unexpectedly, we did not observe increased hypoxia in draining lymph nodes despite evidence of systemic alterations in other tissues. The biological basis for this tissue-specific difference remains unclear and may reflect differences in vascular architecture, immune organization, or metabolic adaptation. Further investigation will be required to determine why draining lymph nodes appear resistant to the hypoxic changes observed elsewhere during PDAC progression.

Few studies have examined the role of hypoxia in promoting Bregs. Meng et al. linked HIF1α to IL10 transcription and showed that mice lacking B cell-specific HIF1a developed more severe collagen-induced arthritis and experimental autoimmune encephalitis (42). Shortly after, Qian et al. demonstrated that HIF1α and pSTAT3 drives CD11b transcription in B cells, such that loss of HIF1α and consequently CD11b led to exacerbated experimental inflammatory bowel disease in mice (57).

An intriguing observation from our study is the disproportionate exposure of tumor-infiltrating B cells to hypoxia compared to other lymphocyte populations. Despite representing a relatively small fraction of tumor-infiltrating lymphocytes, nearly half of intratumoral B cells were hypoxic, and hypoxia intensity appeared greater than that observed in T cells. These findings suggest that B cells either preferentially accumulate within hypoxic tumor niches or possess intrinsic properties that allow them to persist in low oxygen environments. The colocalization of CD19^+^ cells with pimonidazole-positive regions supports the former possibility and aligns with emerging evidence that hypoxic microdomains act as specialized immunoregulatory niches.

Hypoxia-driven immunosuppression has often been linked to adenosine metabolism, particularly through induction of ectonucleotidases such as CD39 and CD73 on regulatory immune populations (58). Our data extend this paradigm to B cells, demonstrating that hypoxic tumor-infiltrating B cells are enriched for dual CD39^+^CD73^+^ expression and exhibit functional capacity to suppress T cell activation via A2A receptor–mediated signaling. Importantly, the enrichment of CD39^+^CD73^+^ B cells was not uniform across tissues, indicating that hypoxia may act in conjunction with tissue-specific or lineage-specific programs to shape regulatory phenotypes. The heightened responsiveness of peritoneal-derived B cells to hypoxia *ex vivo* suggests that baseline metabolic programming and/or developmental origin influences hypoxia sensing and downstream effector functions. Although our findings identify hypoxia as the major driver of the CD39^+^CD73^+^ adenosinergic B-cell phenotype, the tumor microenvironment comprises multiple interconnected metabolic stressors that likely act in concert. Lactate, acidosis, and nutrient deprivation have each been implicated in regulating immune cell function and may further influence ectonucleotidase expression and B-cell phenotype. Our data indicate that lactate modulates, but does not replace, hypoxia-dependent signaling, suggesting that metabolic cues integrate in a context-dependent manner to shape regulatory B-cell states. Future studies will be required to systematically dissect how these individual metabolic stressors cooperate with hypoxia during tumor progression. Thus, a central insight from our work is that hypoxia may differentially regulate regulatory B cell subsets depending on tissue origin.

Specifically, hypoxia reduced IL35 expression and markers of activation in splenic B cells across multiple stimulatory conditions. *In vivo* analyses further showed that hypoxic tumor-infiltrating B cells lacked IL35 expression, supporting the idea that hypoxia actively restrains this regulatory axis rather than simply failing to induce it. This observation contrasts with prevailing models in which hypoxia broadly enhances immunosuppressive programs and instead suggests that different regulatory pathways are preferentially engaged depending on environmental cues (42, 50). One limitation is that we were unable to directly examine IL-35 expression in hypoxic versus non-hypoxic B cells *in vivo*. Spectral overlap between the dual IL-35 reporter system and FITC-conjugated anti-pimonidazole antibody precluded simultaneous quantification, and an alternative single-reporter strategy did not provide sufficient signal for reliable analysis. Consequently, our conclusions regarding hypoxia-mediated suppression of IL-35 rely primarily on ex vivo studies. Development of compatible reporter systems or multiplex imaging approaches will be important for validating these observations directly within the tumor microenvironment.

Conversely, hypoxia enriched CD39^+^CD73^+^ B cells that phenotypically resembled peritoneal-like B cell populations. These cells demonstrated strong association with CD11b expression and were highly enriched among hypoxic tumor-infiltrating B cells. B1 cells are often considered innate-like and metabolically adaptable, characteristics that may render them more capable of surviving and functioning in nutrient-poor or hypoxic environments (59, 60). The preferential expansion or recruitment of peritoneal CD39^+^CD73^+^ B cells within hypoxic tumor niches suggests that hypoxia may drive a shift toward metabolically mediated immunosuppression, relying on adenosine production rather than cytokine-mediated suppression.

Our findings further suggest that IL35 and CD39/CD73 represent partially mutually exclusive regulatory programs. IL35-producing Bregs exhibited minimal ectonucleotidase expression, whereas CD39^+^CD73^+^ populations lacked IL35 expression. Mechanistically, loss of the IL35 subunit Ebi3 enhanced CD39 expression, particularly under hypoxia, and recombinant IL35 treatment reversed this phenotype. These data support a model in which IL35 signaling suppresses alternative immunosuppressive pathways, indicating a degree of regulatory hierarchy or feedback between distinct Breg mechanisms. This reciprocal relationship may represent an adaptive response to environmental constraints. In oxygen-rich environments, cytokine-mediated immunosuppression via IL35 may predominate; however, under hypoxic stress, metabolic pathways that generate extracellular adenosine could become more advantageous. Such functional plasticity would allow B cells to maintain immunosuppressive capacity across heterogeneous tumor microenvironments. Our findings further suggest that regulatory B-cell states should be viewed as preferentially enriched phenotypes rather than mutually exclusive populations. Although splenic tissue-derived B cells are enriched for IL-35 production and peritoneal tissue-derived B cells preferentially express CD39 and CD73, a small degree of overlap exists between these programs. This overlap may reflect phenotypic plasticity, consistent with the dynamic nature of regulatory B-cell differentiation within distinct tissue microenvironments. Accordingly, we have interpreted our findings in the context of tissue-derived populations rather than definitive B-cell lineage assignments.

AB680 has previously demonstrated efficacy in preclinical PDAC models. In a subcutaneous syngeneic KPC model, Faraoni et al. showed that AB680 reduced tumor growth and intratumoral adenosine levels while increasing activated CD8^+^ T cell infiltration (61). The same group later demonstrated that AB680 could prevent PanIN progression in KC genetically engineered mice, reduce immunosuppressive macrophages, and expand unique TCR and BCR clonotypes; single-cell RNA sequencing of these tumors further revealed an increase in mature B cells following AB680 treatment, although immunosuppressive B cell markers were not examined in that study (62). Our findings extend this work by revealing that B cells themselves shape the tumor’s response to CD73 inhibition: the opposing effects of AB680 on tumor volume in wildtype versus B cell-deficient mice, together with the selective reduction of CD39^+^CD73^+^ TILs in wildtype mice, support a model in which B cells constitute a functionally relevant component of the adenosinergic pool in PDAC tumors. The compensatory rise in CD39^+^CD73^+^ peritoneal immune cells in B cell-deficient mice further suggests that non-B populations can partially substitute for B cell-derived adenosine signaling when B cells are absent. However, because AB680 inhibits CD73 broadly across immune and stromal compartments, these experiments cannot fully isolate B cell-intrinsic CD73/adenosine signaling from other cellular sources, and future studies using B cell-specific CD73 or CD39 conditional knockout mice will be required to establish direct causality.

Several questions arise from this work. First, the mechanisms driving preferential localization or survival of peritoneal-like B cells within hypoxic niches remain unclear. Future studies employing lineage tracing or intravital imaging could determine whether these cells migrate from peritoneal reservoirs or undergo local differentiation within tumors. Second, dissecting the transcriptional programs underlying hypoxia-induced CD39/CD73 expression will be critical to understand whether canonical hypoxia-inducible factors directly regulate these pathways in B cells. Third, given the reciprocal relationship between IL35 signaling and adenosine metabolism, it will be important to determine whether therapeutic targeting of one regulatory axis leads to compensatory activation of the other.

From a translational perspective, targeting adenosine signaling may preferentially disrupt hypoxia-adapted Breg populations and enhance anti-tumor immunity. Combination approaches that integrate hypoxia-modulating therapies with checkpoint blockade or adenosine pathway inhibitors could be particularly effective in PDAC, a malignancy characterized by severe hypoxia and resistance to immunotherapy.

In summary, our study identifies hypoxia as a critical determinant of regulatory B cell identity and function in PDAC. Rather than globally enhancing immunosuppressive activity, hypoxia selectively suppresses IL35-producing splenic Bregs while promoting adenosine-generating CD39^+^CD73^+^ peritoneal-like B cells. These findings reveal functional specialization within regulatory B cell subsets and suggest that hypoxic tumor niches shape the dominant mode of B cell-mediated immunosuppression. Understanding how metabolic stress reprograms immune regulatory networks may inform new strategies to overcome immune resistance in pancreatic cancer.

## Data Availability

The original contributions presented in the study are included in the article/**Supplementary Material**. Further inquiries can be directed to the corresponding author.
